# Supplementary material for: Translation, adaptation, validation and performance of the American Weight Efficacy Lifestyle Questionnaire Short Form (WEL-SF) to a Norwegian version: a cross-sectional study
Source: PeerJ. 2014 Sep 16;2:e565. doi: 10.7717/peerj.565 (PMC4178457; doi:10.7717/peerj.565)
Supplement: Supplemental Information 1 [file peerj-02-565-s001.pdf]

Appendix:

American and Norwegian version of the WEL-SF

**WEL-SF Norwegian version**	**WEL-SF American version**
**Jeg er sikker på at:**	**I am confident that:**
1. Jeg kan motstå å spise for mye når jeg er engstelig eller nervøs	I can resist overeating when I am anxious (or nervous)
2. Jeg kan motstå å spise for mye i helgene	I can resist overeating in the weekend
3. Jeg kan motstå å spise for mye når jeg er trøtt	I can resist overeating when I am tired
4. Jeg kan motstå å spise for mye når jeg ser på TV	I can resist overeating when I am watching TV (or use the computer)
5. Jeg kan motstå å spise for mye når jeg er deprimert eller nedstemt	I can resist overeating when I am depressed (or down)
6. Jeg kan motstå å spise for mye når jeg er i sosiale sammenkomster eller på fest	I can resist overeating when I am in a social setting (or at a party)
7. Jeg kan motstå å spise for mye når jeg er sint eller irritabel	I can resist overeating when I am angry (or irritable)
8. Jeg kan motstå å spise for mye når andre presser meg til å spise	I can resist overeating when others are pressuring me to eat

Weight Efficacy Lifestyle Questionnaire Short-Form (WEL-SF)

Read each situation below and decide how confident (or certain) you are that you will be able to resist overeating in each of the difficult situations. On a scale of 0 (not confident) to 10 (very confident), choose ONE number that reflects how confident you feel now about being able to successfully resist the desire to overeat. Write this number next to each item.

0	1	2	3	4	5	6	7	8	9	10
**Not** at all confident										**Very** Confident

**I AM CONFIDENT THAT:**	**Confidence Number**
1. I can resist overeating when I am anxious (or nervous).	_________
2. I can resist overeating on the weekend.	_________
3. I can resist overeating when I am tired.	_________
4. I can resist overeating when I am watching TV (or using the computer).	_________
5. I can resist overeating when I am depressed (or down).	_________
6. I can resist overeating when I am in a social setting (or at a party).	_________
7. I can resist overeating when I am angry (or irritable).	_________
8. I can resist overeating when others are pressuring me to eat.	_________

Ames, G.E., et al., Eating self-efficacy: Development of a short-form WEL, Eating Behaviors (2012), doi: 10.1016/j.eatbeh.2012.03.013.

MESTRINGSFORVENTNING - SPISEVANER

Norsk versjon av "Weight Efficacy Lifestyle Questionnaire Short-Form" (WEL-SF)

(Flølo T.N., et al., 2012)

Les igjennom situasjonene som er beskrevet nedenfor. Besvar deretter spørsmålene om hvor sikker du er på om du vil kunne motstå å spise for mye i hver av disse utfordrende situasjonene. Velg ETT tall på skalaen, fra 0 (ikke sikker i det hele tatt) til 10 (veldig sikker), som beskriver hvor sikker du er på å lykkes med å motstå å spise for mye. Skriv dette tallet etter hvert spørsmål.

0	1	2	3	4	5	6	7	8	9	10
**Ikke** sikker i det hele tatt										**Veldig** sikker

**JEG ER SIKKER PÅ AT:**	**Tall for hvor sikker du er**
1. Jeg kan motstå å spise for mye når jeg er engstelig eller nervøs	_________
2. Jeg kan motstå å spise for mye i helgene	_________
3. Jeg kan motstå å spise for mye når jeg er trøtt	_________
4. Jeg kan motstå å spise for mye når jeg ser på TV	_________
5. Jeg kan motstå å spise for mye når jeg er deprimert eller nedstemt	_________
6. Jeg kan motstå å spise for mye når jeg er i sosiale sammenkomster eller på fest	_________
7. Jeg kan motstå å spise for mye når jeg er sint eller irritabel	_________
8. Jeg kan motstå å spise for mye når andre presser meg til å spise	_________

©Mayo Clinic, Department of Psychiatry and Psychologi: Translated to Norwegian with kind permission of Ames, G.E., 2012
